# Comparative Transcriptome Analysis Provides Novel Insight into Morphologic and Metabolic Changes in the Fat Body during Silkworm Metamorphosis

**DOI:** 10.3390/ijms19113525

**Published:** 2018-11-09

**Authors:** Jian Peng, Zheng Li, Yan Yang, Peng Wang, Xuan Zhou, Tujing Zhao, Mengpei Guo, Meng Meng, Tianlei Zhang, Wenliang Qian, Qingyou Xia, Daojun Cheng, Ping Zhao

**Affiliations:** 1 State Key Laboratory of Silkworm Genome Biology, Southwest University, Chongqing 400715, China; theppone@163.com (J.P.); lzheng92@sina.com (Z.L.); 18404969656@163.com (Y.Y.); 15281715060@163.com (P.W.); zincubus27@gmail.com (X.Z.); 18166571994@163.com (T.Z.); sunx1202@163.com (M.G.); keven190@sina.com (M.M.); zhangtianlei2011@sina.com (T.Z.); wenliang20081103@126.com (W.Q.); xiaqy@swu.edu.cn (Q.X.); chengdj@swu.edu.cn (D.C.); 2 Chongqing Key Laboratory of Sericultural Science, Chongqing Engineering and Technology Research Center for Novel Silk Materials, Southwest University, Chongqing 400715, China

**Keywords:** silkworm, fat body, transcriptome, remodeling, lipid metabolism

## Abstract

The fat body plays key roles in energy storage and utilization as well as biosynthetic and metabolic activities in insects. During metamorphosis from larva to pupa, the fat body undergoes dramatic changes in morphology and metabolic processes. However, the genetic basis underlying these changes has not been completely understood. In this study, the authors performed a time-course transcriptome analysis of the fat body during silkworm metamorphosis using RNA-sequencing. A total of 5217 differentially expressed genes (DEGs) were identified in the fat body at different developmental time points. DEGs involved in lipid synthesis and degradation were highly expressed at the third day of the last larval instar and during the prepupal-pupal transition, respectively. DEGs involved in the ecdysone signaling and bone morphogenetic protein (BMP) signaling pathways that modulate organ development exhibited a high expression level during the fat body remodeling process from prepupa to pupa. Intriguingly, the RNA interference-mediated knockdown of either *decapentaplegic* (*Dpp*) or *protein 60A* (*Gbb*), two DEGs involved in the BMP signaling pathway, inhibited fat body dissociation but promoted lipid mobilization, suggesting that the BMP signaling pathway not only is required for fat body remodeling, but also moderately inhibits lipid mobilization to ensure an appropriate lipid supply during the pupal-adult transition. In conclusion, the comparative transcriptome analysis provides novel insight into morphologic and metabolic changes in the fat body during silkworm metamorphosis.

## 1. Introduction

The fat body is regarded as the center of energy storage and utilization as well as biosynthetic and metabolic activities in insects [1]. During the feeding period, the fat body utilizes nutrients to synthesize hemolymph proteins as well as storage proteins and to produce energy reserves of fat and glycogen [2,3]. During the metamorphotic period with the larval-pupal transition, the fat body provides the necessities for survival, including energy and amino acids [4].

Generally, the fat body undergoes dramatic morphological and functional alterations during insect metamorphosis from larva to pupa and this event is called fat body remodeling [5]. Morphologically, the larval fat body undergoes tissue dissociation and separates into individual fat cells [6]. Several genes that respond to the steroid hormone ecdysone, which is involved in the initiation of larval molting and metamorphosis [7,8], have been demonstrated to play key roles in fat body remodeling [9,10,11]. For example, in *Drosophila*, matrix metalloproteinase 1 (Mmp1) and matrix metalloproteinase 2 (Mmp2), two direct targets of the ecdysone-responsive fushi tarazu transcription factor 1(Ftz-f1), promote fat cell dissociation [10,12,13]. Knockdown of either *Mmp1* or *Mmp2* delays fat body cells dissociation [13]. Consequently, the disruption of fat body remodeling leads to a failure of the larval-pupal transition [14]. However, little is known about the genome-wide response to structural alterations of the fat body during insect metamorphosis.

The fat body functions as a metabolic center during insect growth and development [1]. Insect fat body cells are reserved in the abundant lipid storage in the form of triacylglycerol (TAG). The lipid droplet is a specific cytoplasmic organelle that functions in the storage of TAG, and it undergoes periodic changes in its size as the result of lipolysis or lipogenesis [15]. Specifically, the intermediary metabolic processes in the fat body mainly include lipid and carbohydrate metabolism [16,17], protein synthesis [18], and amino acid and nitrogen metabolism [19]. As is well known, the insulin/insulin-like growth factor signaling (IIS) and target of rapamycin (TOR) signaling pathways are primarily involved in the development and functions of the insect fat body by maintaining metabolic homeostasis [20]. Reduced TOR activity leads to non-phosphorylated forkhead box protein O (FoxO) enriched in the nucleus and enhances the expression level of the eukaryotic translation initiation factor 4E-binding protein (*4E-BP*)gene involved in lipid mobilization [21,22,23]. Additionally, inappropriate changes in the structure of the fat body can cause metabolic disorders during the fat body remodeling process [24,25].

Although increasingly more studies have illustrated physiological processes and signaling pathways in the insect fat body, the genetic basis underlying the morphological and functional changes of the fat body during insect metamorphosis have not been completely understood at the transcriptome level. In this study, the authors used the silkworm (*Bombyx mori*) as a model to analyze transcriptome changes in the fat body during metamorphosis via the RNA-seq method and obtained a more comprehensive insight into the relationship between the remodeling and metabolic changes during development in the silkworm fat body.

## 2. Results

### 2.1. Morphological Dynamic Changes of the Fat Body and Lipid Droplets during Silkworm Metamorphosis

**Figure 1:**
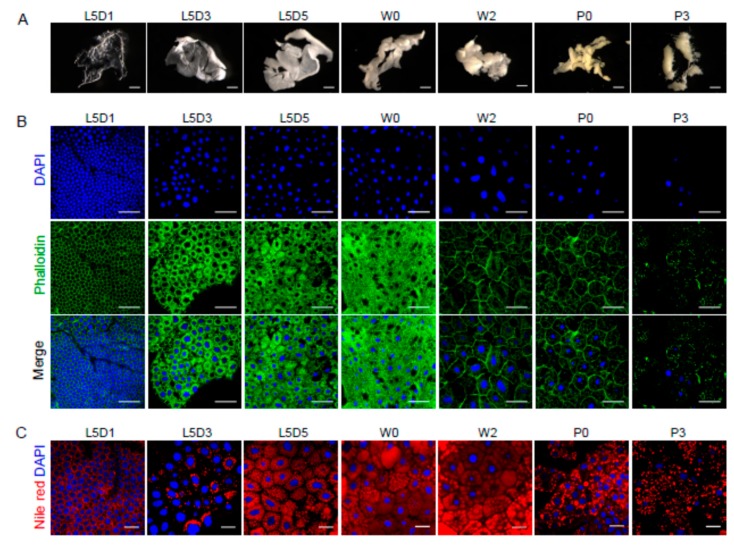
Morphological changes of cells and lipid droplets in the fat body during silkworm metamorphosis. (**A**) Dynamic changes in the morphology of the silkworm fat body. (**B**) Developmental changes in the cell shape of silkworm fat body cells. (**C**) Morphological changes of lipid droplets during the silkworm fat body remodeling. L5D1, the first day of the last larval instar; L5D3, the third day of the last larval instar; L5D5, the fifth day of the last larval instar; W0, just wandering; W2, the second day during wandering; P0, just pupation; P3, the third day after pupation. Blue, DAPI (nucleus); Green, Phalloidin (actin); Merge, DAPI and Phalloidin; Red, Nile red (lipid). Scale bars: (**A**) 2 mm, (**B**) 50 μm, (**C**) 20 μm.

To better understand the mechanism underlying the functional changes in the fat body that occur during silkworm metamorphosis, the authors first investigated the morphological diversity of the fat body and lipid droplets at seven time points during the larval-pupal transition, including the first day of the last larval instar (L5D1), the third day of the last larval instar (L5D3), the fifth day of the last larval instar (L5D5), just wandering (W0), the second day during wandering (W2), just pupation (P0), and the third day after pupation (P3). Time-course analysis revealed that, at L5D1, the silkworm fat body was composed of transparent layers that were closely connected to the trachea (Figure 1A). 

During silkworm larval development from L5D1 to L5D5, the fat body increased in volume and became a thick layer. At the beginning of the wandering (W0), which is accompanied by the cessation of feeding, the fat body layers gradually retracted. From the second day during wandering (W2) to pupa (P3), the fat body cells began to be disaggregated and detached, and the fat body was transformed into particles. However, the silkworm fat body could not completely separate because of its connection with the trachea, which is somewhat different from the fat body in *Drosophila melanogaster* [6]. Fluorescence staining also showed that the fat body cells gradually increased in size and tightly adhered to each other during the feeding stage of the fifth larval instar, and then the cells were dissociated from each other during the larval-pupal transition (Figure 1B). 

Generally, the lipid droplet in cells contains neutral lipids, mainly TAG and cholesteryl esters, and is essential for energy storage [26,27,28]. Using Nile red staining, the authors found that lipid droplets were more abundant from L5D5 (late stage of last larval instar) to W2 (before pupation), and their abundance significantly reduced after pupation (Figure 1C), indicating that lipid mainly accumulated in the late stages of the last larval instar and was utilized after prepupation.

### 2.2. RNA-Seq Analysis of the Silkworm Fat Body Transcriptomes

**Figure 2:**
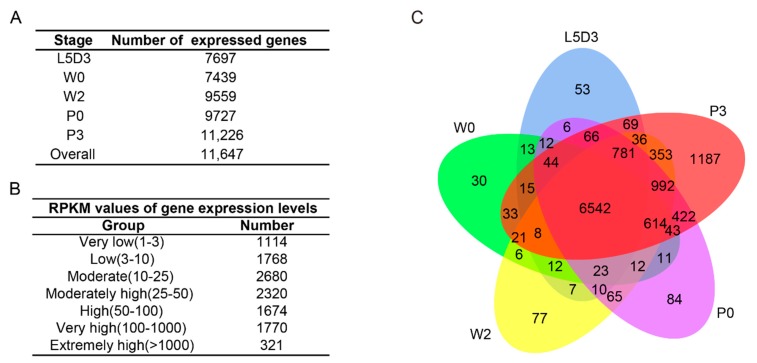
Transcriptome sequencing and gene expression profiling of the fat body at different stages during silkworm metamorphosis. (**A**) Number of genes expressed in the silkworm fat body at different developmental stages. (**B**) RPKM (reads per kilobase per million mapped reads) values of gene expression levels. (**C**) Venn plot analysis of the genes expressed in the silkworm fat body at each developmental stage. Blue, L5D3; Green, W0; Yellow, W2; Violet, P0; Red, P3; L5D3, the third day of the last larval instar; W0, just wandering; W2, the second day during wandering; P0, just pupation; and P3, the third day after pupation.

In order to identify the gene signature that contributes to the dynamic changes of both the morphology and functions of the fat body during silkworm metamorphosis, the authors performed an RNA-seq analysis of the fat body at five time points including L5D3, W0, W2, P0, and P3. In total, their RNA-seq analysis generated 178,419,240 raw reads and 173,776,559 clean reads (Table S1). Pearson correlation analysis revealed that the repeated samples had a perfect consistency (Figure S1). Further analysis identified that a total of approximately 90% of the reads were mapped to the silkworm reference genome and were mapped to 11,647 genes, including 7697 for L5D3, 7439 for W0, 9559 for W2, 9727 for P0, and 11,226 for P3 (Figure 2A). In addition, based on the RPKM (reads per kilobase per million mapped reads) values for the expressed genes, the authors observed that 3765 genes showed a high expression as indicated by RPKM values over 50 (Figure 2B). Further analysis found that 53, 30, 77, 84, and 1187 genes were specifically expressed at L5D3, W0, W2, P0, and P3, respectively (Figure 2C). 

### 2.3. Inventory of Differentially Expressed Genes (DEGs) in the Silkworm Fat Body among Different Developmental Stages

**Figure 3:**
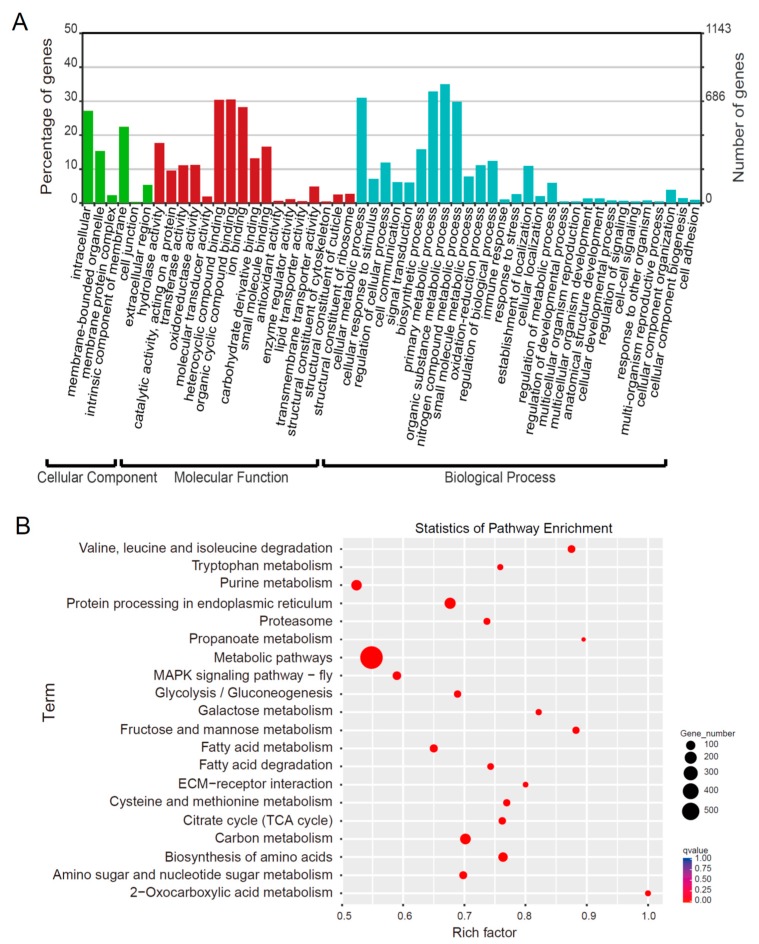
Gene ontology (GO) and Kyoto Encyclopedia of Genes and Genomes (KEGG) pathway enrichment analyses of the differentially expressed genes (DEGs) in the fat body at different stages during silkworm metamorphosis. (**A**) GO annotation of all DEGs. Green, Cell Component; Red, Molecular Function; Blue, Biological Process. (**B**) Scatter plot of the enriched KEGG pathways for all DEGs. Size of dots, Number of enriched genes; Color of dots, the red dots indicated pathways with significant enrichment and the blue dots indicated pathways with low enrichment; Rich factor = the DEGs number/ the number of genes in this pathway; MAPK: mitogen-activated protein kinase; ECM: extracellular matrix; TCA: tricarboxylic acid.

A comparative analysis indicated that 5217 genes were differentially expressed in the fat body during silkworm metamorphosis (Table S2), which are called differentially expressed genes (DEGs). A gene ontology (GO) annotation analysis showed that the number of DEGs belonging to the categories of binding and catalytic activity in molecular function and metabolic and cellular processes in biological processes were the largest (Figure 3A). Similarly, a Kyoto Encyclopedia of Genes and Genomes (KEGG) analysis also revealed that the most DEGs were related to basic metabolism, protein processing in endoplasmic reticulum, and amino acid biosynthesis (Figure 3B). 

**Figure 4:**
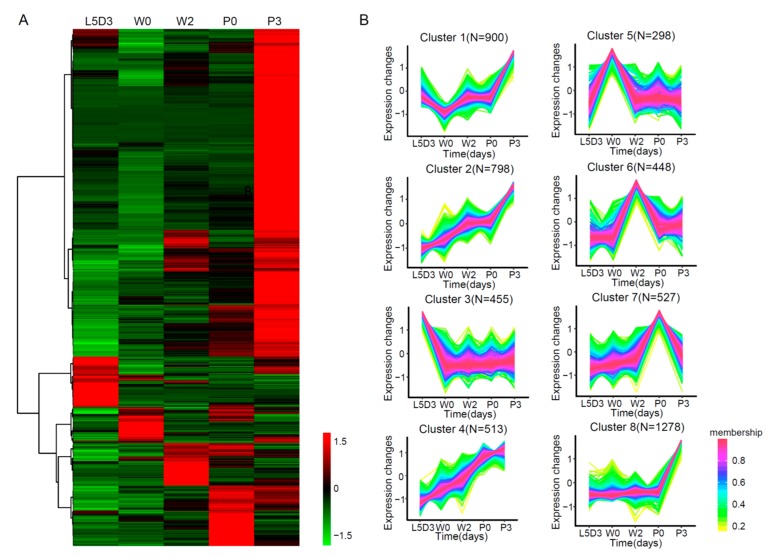
Clustering analysis of all DEGs in the silkworm fat body. (**A**) Hierarchical clustering of all DEGs. Red, genes with high expression levels; Green, gene with low expression levels. (**B**) Co-expression analysis of all DEGs. The digits denote the number of genes in each cluster. The DEGs in each cluster are listed in Table S3. Red, the high correlation between genes; yellow, the low correlation between genes.

A hierarchical clustering analysis showed that these DEGs mainly exhibited stage-specific expression in the fat body during silkworm metamorphosis (Figure 4A and Table S2). A quantitative real-time polymerase chain reaction (RT-PCR) examination confirmed that the expression changes of several selected DEGs showed similar changes as those in the RNA-seq data (Figure S2). Intriguingly, the number of DEGs that were highly expressed at the P3 stage was the largest, indicating that there was a dramatic physiological change in the fat body at this stage. Further co-expression analysis grouped these DEGs into eight clusters (Figure 4B and Table S3). DEGs from cluster 3 were highly expressed at L5D3. DEGs from clusters 6 and 7 showed a high expression during prepupa (W2 to P0). DEGs from four clusters (clusters 1, 2, 4, and 8) were highly expressed at P3.

Notably, most of the 455 DEGs that were highly expressed at L5D3 were enriched in ion binding, oxidoreductase activity, heterocyclic compound binding, and organic cyclic compound binding. Interestingly, two kinds of genes with the highest expression at L5D3, storage proteins and chymotrypsin inhibitors, had a nutrient reservoir activity and a protein degradation inhibition activity, respectively, indicating that L5D3 is a stage for the storage of nutrients and the inhibition of protein hydrolysis. In contrast, 3489 DEGs had a higher expression at P3, most of them being associated with organic cyclic compound binding, heterocyclic compound binding, ion binding, and hydrolase activity, indicating that nutrient utilization and cell biosynthesis processes mainly occurred in the fat body at P3 (Table S4). Furthermore, the fat body began to be dissociated and the extracellular matrix was remodeled during silkworm prepupal stages from W0 to P0. The study’s analysis uncovered that in addition to DEGs involved in ecdysone signaling, DEGs that were annotated with the extracellular region term, which may be associated with tissue dissociation and remodeling, were highly expressed during prepupal stages: for example, extracellular matrix (matrix metalloproteinase 1, Mmp1; matrix metalloproteinase 2, Mmp2; type IV collagen), extracellular space (serine protease inhibitor 3, serine protease inhibitor 5, serine protease inhibitor 13, serine protease inhibitor 28, antitrypsin), and others of the extracellular region (decapentaplegic, glycoprotein, type IV collagen, and apolipophorin-III). The analysis also included cell adhesion term (integrin alpha-position-specific 1, integrin alpha-IIb, integrin beta subunit 1, BMP—binding endothelial regulator protein, integrin alpha—position-specific 3) (Table S5).

### 2.4. DEGs Involved in Lipid Metabolism in the Fat Body during Silkworm Metamorphosis

**Figure 5:**
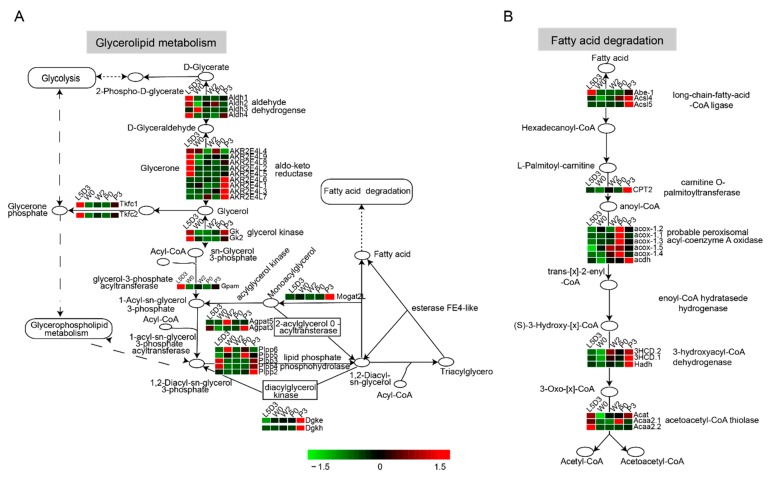
DEGs involved in glycerolipid metabolism and fatty acid degradation pathway. (**A**) Expression changes of DEGs involved in glycerolipid metabolism. (**B**) Expression changes of DEGs involved in fatty acid degradation. Red, genes with high expression levels; Green, gene with low expression levels; Rounded square frame: KEGG pathway names; square frame: enzymes; oval: intermediate product; black arrows: direction of metabolic pathway; L5D3, the third day of the last larval instar; W0, just wandering; W2, the second day during wandering; P0, just pupation; P3, the third day after pupation. DEGs involved in glycerolipid metabolism and fatty acid degradation pathways are listed in Table S6. Sn: stereo-specifically numbered; D:dextrorotation; L: laevorotation; O: oxidation; CoA: coenzyme A.

**Figure 6:**
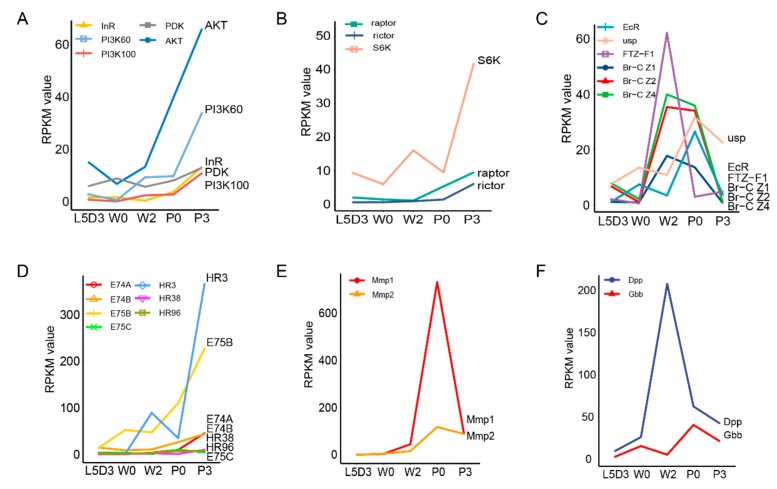
Expression changes of DEGs involved in several signaling pathways related to metabolic activity and organ remodeling. (**A**,**B**) DEGs involved in the insulin/insulin-like growth factor signaling (IIS) and target of rapamycin (TOR) signaling pathways, which are related to metabolic activity. (**C–F**): (**C**,**D**) DEGs involved in the ecdysone signaling pathway, (**E**) matrix metalloproteinases, and (**F**) the bone morphogenetic protein (BMP) signaling pathway, which are related to organ remodeling. AKT, serine/threonine protein kinase; InR, insulin receptor; PDK, pyruvate dehydrogenase kinase; usp, ultraspiracle protein; EcR, ecdysone receptor; FTZ-F1, fushi tarazu transcription factor 1; HR, hormone receptor; Mmp, matrix metalloproteinase; Dpp, decapentaplegic; Gbb, protein 60A; L5D3, the third day of the last larval instar; W0, just wandering; W2, the second day during wandering; P0, just pupation; P3, the third day after pupation.

Lipid is the main component of the fat body, in which fatty acids are converted into triglycerides. The KEGG analysis showed that the pathways directly involved in lipid metabolism were significantly enriched, such as glycerolipid metabolism and fatty acid degradation (Figure 5 and Table S6). As is well known, triacylglycerol biosynthesis involves the monoacylglycerol pathway, the dihydroxyacetone phosphate pathway, and the sn-glycerol-3-phosphate pathway. The authors noted that genes belonging to the dihydroxyacetone phosphate and sn-glycerol-3-phosphate pathways were highly expressed at L5D3 and P3, and the genes involved in the monoacylglycerol pathway exhibited a high expression at P3 (Figure 5A). In addition, most of the genes that associated with the insulin pathway, including insulin receptor (*InR*), phosphoinositide 3- kinase 100 (*PI3K100*), phosphoinositide 3- kinase 60 (*PI3K60*), and pyruvate dehydrogenase kinase (*PDK*) and the TOR pathway, including regulatory-associated protein of TOR (*raptor*), rapamycin-insensitive companion of mTOR (*rictor*), and ribosomal protein S6 kinase (*S6k*), which are involved in maintaining metabolic homeostasis, were up-regulated at P3 (Figure 6A,B). This indicated that lipid synthesis was active at L5D3 and P3.

Fatty acids are mobilized from the fat body for use by other tissues either as an energy source or for other purposes by other tissues. This process released from the triacylglycerols is sequentially driven by various lipases, including adipose triacylglycerol lipase (ATGL), triglyceride lipase (TGL), hormone-sensitive lipase (HSL), and monoacylglycerol lipase. In the study’s data, *TGL*, *HSL*, and monoacylglycerol lipase were highly expressed at P3, but the expression of *ATGL* was low at this stage (Table S7). Moreover, DEGs involved in fatty acid degradation were highly expressed during the prepupal-pupal transition (Figure 5B). These data are consistent with the requirement of energy for the prepupal-pupal transition.

### 2.5. DEGs Involved in the Fat Body Remodeling during Silkworm Metamorphosis

Fat body remodeling, mainly including fat body dissociation, occurs during insect metamorphosis and is required for the lipid mobilization. The authors’ analysis revealed that as expected, DEGs involved in the ecdysone signaling pathway, which has been demonstrated to control insect fat remodeling, exhibited a high expression during the prepupal-pupal transition, including ecdysone receptor (*EcR*), broad-complex (*Br-C*), ecdysone-induced protein 75B (*E75B*), ecdysone-induced protein 74A (*E74A*), ecdysone-induced protein 74B (*E74B*), ecdysone-induced protein 75C (*E75C*), and fushi tarazu transcription factor 1 (*Ftz-f1*) (Figure 6C,D). In addition, as mentioned above, *Mmp1* and *Mmp2*, two genes encoding matrix metalloproteinases that are related to extracellular matrix and involved in tissue remodeling, were highly expressed during the transition from prepupa to pupa (Figure 6E). 

Interestingly, the authors also observed that some DEGs related to specific developmental pathways showed significant alternations in the fat body during silkworm metamorphosis. For example, the expression of some genes associated with the BMP signaling pathway were significantly altered from W2 to P0. *Dpp* was highly expressed at W2 and *Gbb* was up-regulated after wandering (Figure 6F). It showed that the BMP signal was highly active during metamorphosis.

### 2.6. Downregulation of Either Dpp or Gbb Inhibits Morphological Alteration and Promotes Lipid Mobilization in the Fat Body during Silkworm Metamorphosis

**Figure 7:**
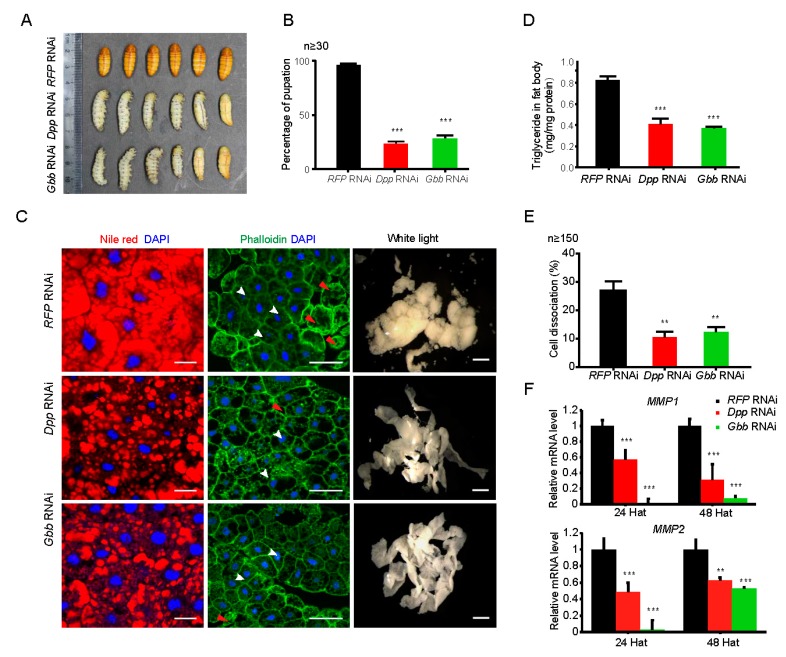
RNA interference-mediated knockdown of either *decapentaplegic* (*Dpp*) or *protein 60A* (*Gbb*) affected fat body remodeling and lipid mobilization during silkworm metamorphosis. (**A**,**B**) Knockdown of either *Dpp* or *Gbb*, two genes involved in the BMP signaling pathway, blocked silkworm pupation. (**C**) Knockdown of either *Dpp* or *Gbb* inhibited fat body remodeling and promoted lipid mobilization during silkworm metamorphosis. White arrow: regular cells; Red arrow: isolated cells. Blue, DAPI (nucleus); Green, Phalloidin (actin); Red, Nile red (lipid). Scale bars: Nile red/DAPI and Phalloidin, 50 μm; white light, 2 mm. (**D**) Triacylglycerol (TAG) level in the silkworm fat body was decreased after knockdown of either *Dpp* or *Gbb*. (**E**) Cell dissociation in silkworm fat body decreased after the RNA interference (RNAi)-mediated knockdown of either *Dpp* or *Gbb*. Cell dissociation (%) = The number of dissociated cells/the number of total cells × 100. (**F**) Knockdown of either *Dpp* or *Gbb* reduced the expression of the matrix metalloproteinase (*Mmp*) genes in silkworm fat body. Hat, hours after treatment. All experiments were performed in three biological replicates. Values are represented as the mean ± standard error (error bars). For the significance test: ** *p* < 0.01, *** *p* < 0.001 vs. control.

Fat body remodeling occurs during the early stages of silkworm metamorphosis, and metabolic processes are also altered during the fat body remodeling. To investigate the relationship between morphological alterations and lipid mobilization in the silkworm fat body, the authors further performed an RNA interference (RNAi)-mediated knockdown of *Dpp* and *Gbb*, two development-related genes involved in the BMP signaling pathway that were highly expressed during silkworm pupation. The double-stranded RNA oligonucleotides with silencing activity double-stranded RNA oligonucleotides with silencing activity against *Dpp* or *Gbb* was injected into silkworm individuals at 6 h after just wandering. At 24 h after injection, the expression of *Dpp* and *Gbb* in the fat body was decreased by 65% and 33.4%, respectively, compared with the red fluorescent protein (*RFP*) gene RNAi as the control (Figure S3A). Moreover, the authors observed that compared with the control, the RNAi-mediated knockdown of either *Dpp* or *Gbb* caused a delay in pupation, with 23.3% or 30%, respectively (Figure 7A,B). It showed that either *Dpp* or *Gbb* both have key roles in silkworm metamorphosis.

The authors further analyzed the effects of the knockdown of either *Dpp* or *Gbb* on lipid mobilization and cell dissociation in the fat body during silkworm metamorphosis. As shown in Figure 7C, Nile red staining revealed that lipid storage was dramatically attenuated following the knockdown of either the *Dpp* or *Gbb* gene. TAG level was also substantially reduced after the knockdown of either *Dpp* or *Gbb* (Figure 7D). Additionally, yip2 (yippee interacting protein 2), a carnitine acyl transferase and lip3 (lipase 3), the lipid droplet-associated protein, were up-regulated after *Dpp* or *Gbb* knockdown (Figure S3B). Moreover, the knockdown of either *Dpp* or *Gbb* significantly inhibited the dissociation of fat body cells (Figure 7C). The rate of cell dissociation also decreased after the RNAi-mediated knockdown of either *Dpp* or *Gbb* (Figure 7E). Moreover, both *Mmp1* and *Mmp2*, two genes involved in tissue dissociation [13], were also down-regulated after *Dpp* or *Gbb* knockdown (Figure 7F). These results suggested that either *Dpp* or *Gbb* inhibits lipid degradation and promotes cell dissociation.

## 3. Discussion

In this study, the authors characterized the dramatic changes in morphology, lipid droplet size, and gene expression that occur in the fat body during silkworm metamorphosis from last larval instar to pupa. First, they observed that the sizes of fat body cells and lipid droplets gradually increased during larval development, indicating an abundant accumulation of nutrition and energy. However, the fat body remodeling following with cell dissociation and lipid droplet degradation obviously occurred during the prepupal-pupal transition, indicating a dramatic mobilization of nutrients and energy. These changes are similar to the previously reported changes in the *Drosophila* fat body [6]. Notably, the distributions of trachea caused some layers of fat body cells not to be completely detached from the trachea at the pupa stage in the silkworm, which is a notable difference from the orthologous process in *Drosophila* where the layers of the fat body cells dissociate into individual fat cells during the larval to pupal stages [6]. 

Glycerolipid metabolism and fatty acid degradation are regarded as two important pathways involved in the synthesis and mobilization of lipid [29,30]. The authors’ analysis about time-course changes of the transcriptomes in the fat body during silkworm metamorphosis revealed that the pathways controlling TAG storage and utilization, including the glycerolipid metabolism pathway as well as the insulin/insulin-like and TOR signaling pathways, were significantly up-regulated at the third day of the last larval instar and the third day after pupation. Otherwise, genes involved in fatty acid degradation were highly expressed during the prepupal-pupal transition. A previous study also demonstrated that lipogenesis is a core-dominated progress in the first half of the final stage in the silkworm [31]. Taking the above points together, the authors propose that lipid metabolism is important for nutritional supply during the remodeling of the silkworm fat body.

As is well known, ecdysone triggers insect metamorphosis [32]. In fact, ecdysone has been demonstrated to control fat body cell dissociation during metamorphosis via Mmp proteins and cathepsin L [10,33], and to modulate systematic insulin/insulin-like growth factor signal and body growth by regulating fat body-expressed imaginal morphogenesis protein-Late 2 (Imp-L2), a *Drosophila* homolog of insulin-like growth factor-binding protein 7 [34]. The authors observed that genes involved in the ecdysone signaling pathway, including *Ftz-F1* and *Br-C*, were up-regulated at the prepupal stages from the second day after wandering to just pupation. The expression of *Mmp1* and *Mmp2* was also increased during metamorphosis. When these points are taken together, it is clear that ecdysone controls both the remodeling and the metabolic changes in the fat body.

An interesting finding is that the BMP signaling pathway seemed to initiate the remodeling but inhibit lipid mobilization in the silkworm fat body. Previous studies reported that the BMP signaling pathway is involved in embryogenesis, imaginal disc development, oogenesis, and maintenance of proper metabolism [35,36], and if the *Gbb*, one of two *Drosophila* orthologues of vertebrate BMP, is missing, it causes a decrease in lipid stores [36]. The authors’ results found that *Gbb* and *Dpp*, which is another *Drosophila* orthologue of vertebrate BMP, displayed a high expression in prepupa, which is similar to the expression profiles of *Mmp1* and *Mmp2*. The knockdown of either *Gbb* or *Dpp* delayed fat body remodeling but reduced the density of lipid droplets and the expression of *Mmp*s in the fat body. The authors’ findings, together with previous reports, revealed that the BMP signal plays dual roles in the fat body and that its moderate inhibition on lipid mobilization may contribute to ensuring lipid mobilization during the pupal-adult transition. This is different from the roles of ecdysone in regulating the remodeling and lipid mobilization in the insect fat body. It is worthy to explore whether ecdysone can interact with the BMP signaling pathway in insects.

## 4. Materials and Methods 

### 4.1. Silkworm Strain

Silkworm strain Dazao (p50) were raised on fresh mulberry leaves, under the regular conditions of temperature of 25 °C and relative humidity of 70–80%, in the professional room in the State Key Laboratory of Silkworm Genome Biology. Silkworm fat bodies were dissected from individual silkworms at different time points (L5D1, L5D3, L5D5, W0, W2, P0, P3, as defined in Section 2.1). The samples were prepared from male larvae. For each sample, one part of the samples was fixed in 4% paraformaldehyde, and the other part of the samples was placed in liquid nitrogen and stored at −80 °C until use.

### 4.2. Morphological Observation of the Silkworm Fat Body

Fat body organs from silkworm larvae were collected at the indicated times and then fixed in 4% paraformaldehyde for 30 min at room temperature. After fixation, the samples were washed three times with phosphate-buffered saline (PBS). The washed fat body samples were incubated with Nile Red (1:1000, Sigma-Aldrich, St. Louis, MO, USA), DAPI (1:1000, Life Technologies, Carlsbad, CA, USA) and phalloidin (1:500, Life Technologies, Eugene, OR, USA). Fluorescence signals were captured by confocal microscopy (Olympus, Tokyo, Japan) at the excitation wavelengths of 488 and 594 nm. White light signals were captured using an M165FC microscope (Leica Microsystems, Wetzlar, Germany).

### 4.3. RNA Sequencing

For RNA sequencing, total RNA was extracted from fat body samples acquired at different developmental stages respectively. Fat bodies samples (1–10 mg) were dissected in PBS and directly transferred to TRIzol^®^ reagent (Invitrogen, Carlsbad, CA, USA), and then ground with liquid nitrogen. RNA was extracted again using trichloromethane, precipitated using isopropanol and cleared using 75% ethanol. The RNA samples were stored in a refrigerator at −80 °C for sequencing libraries. Each cDNA sample was clustered using Oligo-dT magnetic beads and then sequenced on the HiSeq2000 platform (Illumina, San Diego, CA, USA) using single-read technology. All of the raw data presented in this study have been deposited in the National Center for Biotechnology Information (NCBI) Short Read Archive (http://www.ncbi.nlm.nih.gov/sra/) under the Sequence Read Archive (SRA) accession number (SRP156111). 

### 4.4. Data Analysis

The silkworm reference genome was downloaded from the SilkDB2.0 (http://www.silkdb.org/silkdb/). Clean data were mapped to the silkworm transcriptome reference database using Tophat2 [37]. Only data uniquely mapping to reference sequences reads were retained for subsequent analyses. Read count data were obtained using HTseq [38]. Gene expression values were calculated using the RPKM (Reads Per kb per Million reads) method. All expression genes with RPKM ≥ 1 were annotated using Blast2go software (http://www.blast2go.com/b2ghome). For each compared group, DEGs were detected by DESeq [39] with |log2(FoldChange)| > 1 and qvalue < 0.005, including the transcripts of W0, which were compared with those of L5D3 (L5D3 as control); the transcripts of W2, compared with those of W0 (W0 as control); the transcripts of P0, compared with those of W2 (W2 as control); and the transcripts of P3, compared with those of P0 (P0 as control). Based on the Web Gene Ontology Annotation Plot (WEGO) online program, the authors functionally analyzed the GO terms of DEGs. Then, the KEGG pathway database (http://www.genome.jp/kegg/) enrichment analysis was conducted using KOBAS3.0 (http://kobas.cbi.pku.edu.cn/). The hierarchical clustering of gene expression was performed using the pheatmap [40]. To analyze parallel gene expression changes of the differentially expressed genes across five time points in silkworm, the authors performed a co-expression analysis using TCseq [41].

### 4.5. Quantitative RT-PCR Analysis

Each quantitative RT-PCR (qRT-PCR) reaction was conducted using a final volume of 20 µL, which contained 10 µL of SYBR^®^, 6.4 µL of ddH_2_O, 1.6 µL (0.8 µM) of primers, and 2 µL (70 ng) of cDNA. qRT-PCR was performed with the SYBR^®^ Premix Ex Taq™ system (TaKaRa Biotech, Kusatsu, Japan), using the following conditions which were set as 95 °C for 30 s, followed by 40 cycles of 95 °C for 3 s, 60 °C for 30 s. The ribosomal protein L3 (*Rpl3*) gene was used as the endogenous control. All primers used in this study are listed in Table S8. The Pearson correlation coefficient between the RNA sequencing (RNA-seq) data and the qRT-PCR results of DEGs was further calculated.

### 4.6. Triacylglycerol (TAG) Measurements

For TAG assays, six male silkworm fat body tissues were mixed. According to the manufacturer’s instructions, each sample was homogenized in 350 μL of 5% Nonidet P-40, heated at 70 °C for 5 min, centrifuged at 13,000 rpm for 10 min at 4 °C, and 10 μL supernatants was used to measure triglycerides (Triglyceride Determination Kit, Sigma-Aldrich, St. Louis, MO, USA) or protein concentrations (Enhanced BCA Protein Assay Kit, Beyotime, Shanghai, China). TAG levels were normalized to that of protein levels.

### 4.7. Injection of Double-Stranded RNA (dsRNA)

Double-stranded RNA (dsRNA) was generated for *RFP*, *Dpp*, and *Gbb* using the T7 RiboMAXTM™ Express RNAi System (Promega, Madison, WI, USA) according to the manufacturer’s instructions. All dsRNA primers used in this study are listed in Table S8. RNAi injection was administered at 6 h after just wandering (W0). Fat body tissues were collected for bioassays at 24 h and 48 h after the injection of 20 μg dsRNA. The number of each silkworm group was 30, and three biological replicates were executed. 

### 4.8. Statistical Analysis

Statistical analysis was performed using the Student’s *t*-test in this study. Data are presented as the mean ± standard error of three independent biological replicates, and *p* < 0.05 was accepted as significant. * indicates *p* < 0.05; ** indicates *p* < 0.01; *** indicates *p* < 0.001.

## Supplementary Materials

Supplementary materials can be found at http://www.mdpi.com/1422-0067/19/11/3525/s1.

## Abbreviations

DEG	Differentially expressed gene
TAG	Triacylglycerol
TOR	Target of rapamycin
GO	Gene ontology
Hat	Hours after treatment
