# Supplementary figures and images for: Comparative Transcriptome Analysis Provides Novel Insight into Morphologic and Metabolic Changes in the Fat Body during Silkworm Metamorphosis
Source: Int J Mol Sci. 2018 Nov 9;19(11):3525. doi: 10.3390/ijms19113525 (PMC6274779; doi:10.3390/ijms19113525)

Pearson correlation between samples

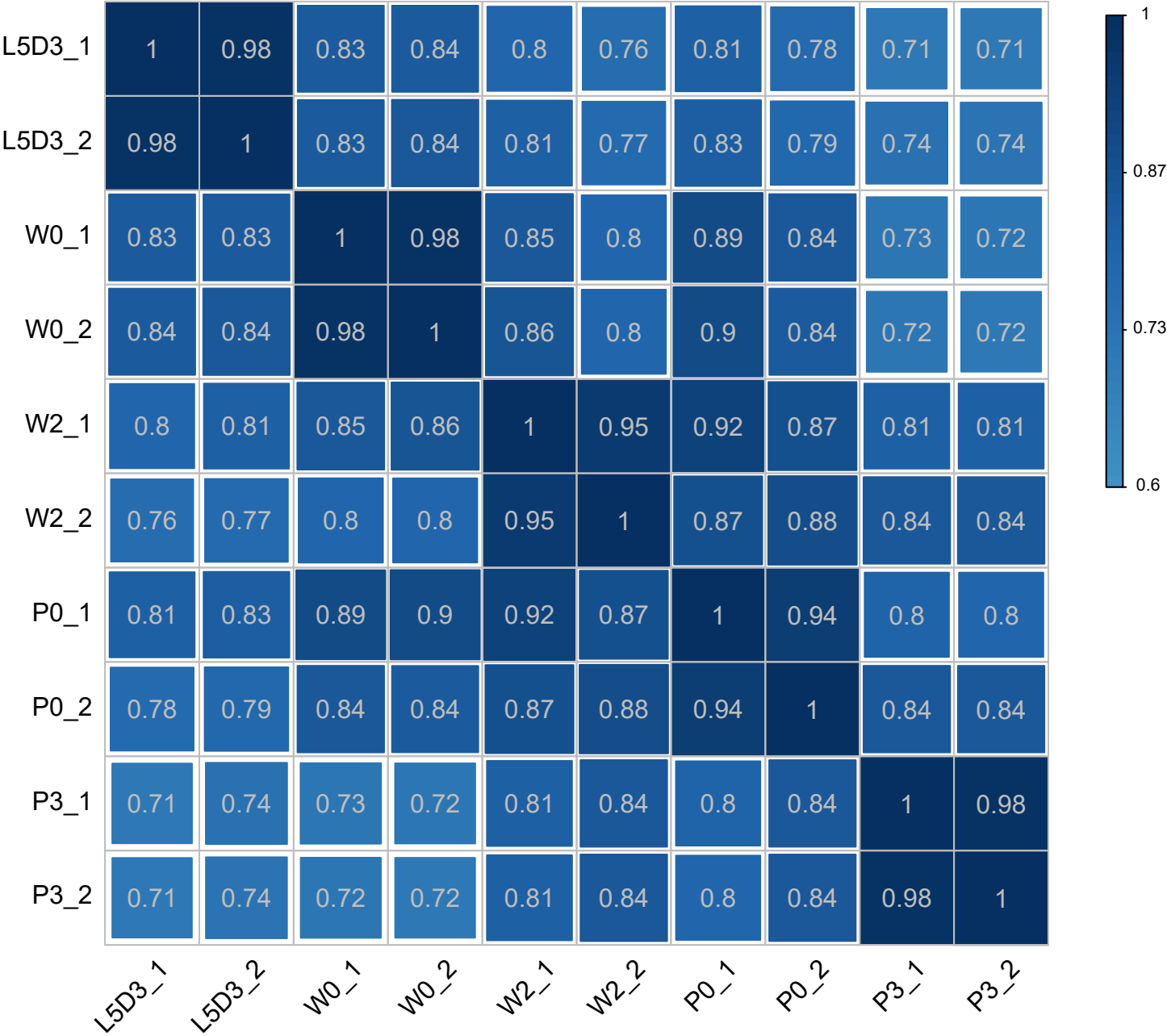

Supplement: Supplementary file 1 [file ijms-19-03525-s001.zip › Figure S1. Pearson correlation between samples.pdf]

A

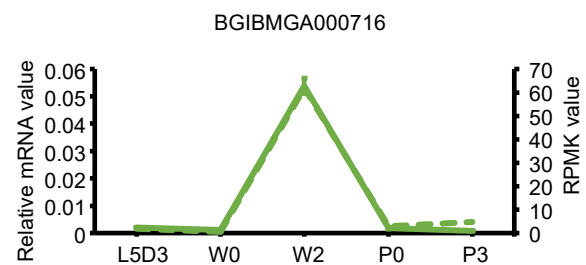

B

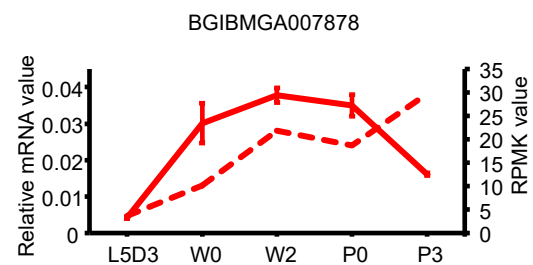

C

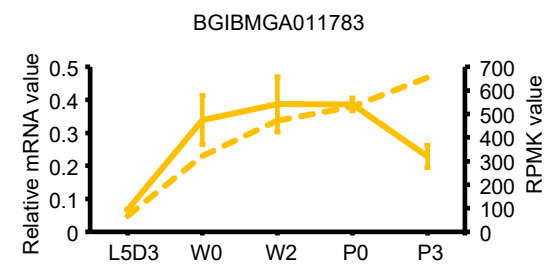

D

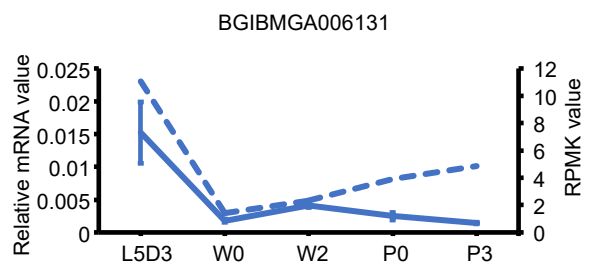

E

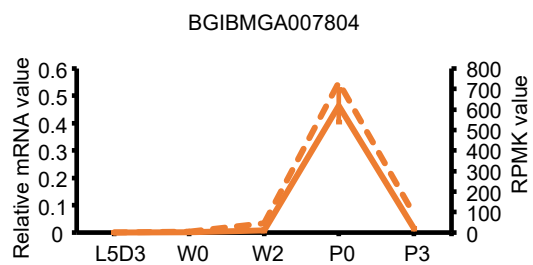

F

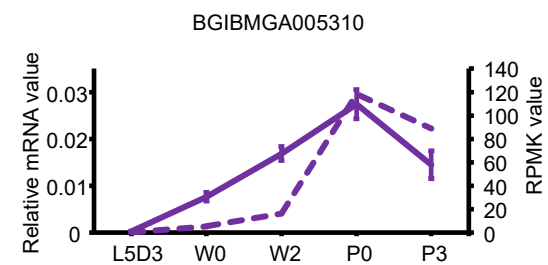

G

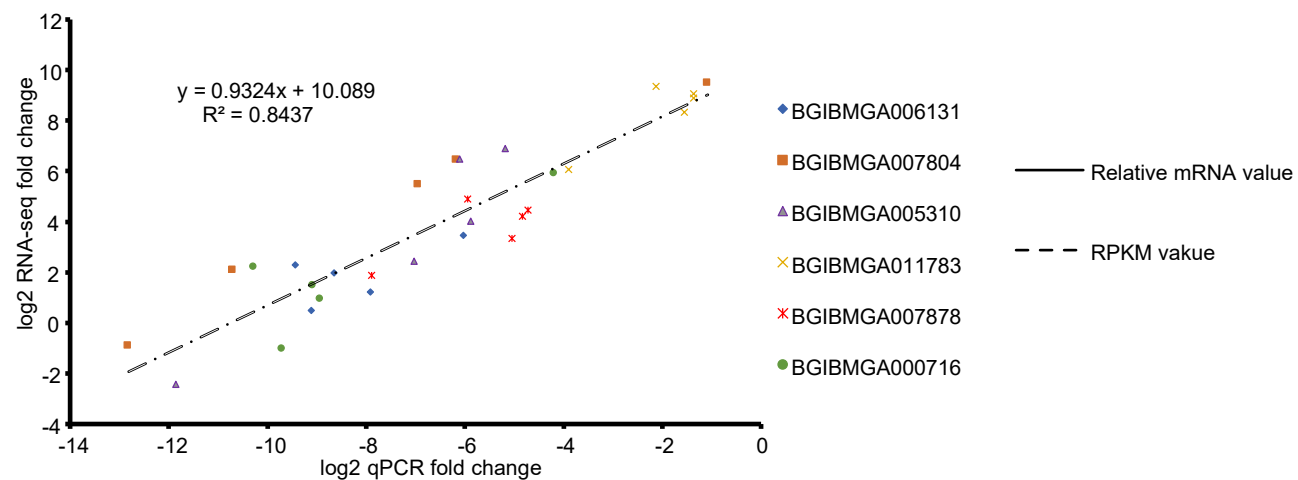

Supplement: Supplementary file 1 [file ijms-19-03525-s001.zip › Figure S2. Transcriptomic and qPCR analysis of the genes in DEGs.pdf]

A

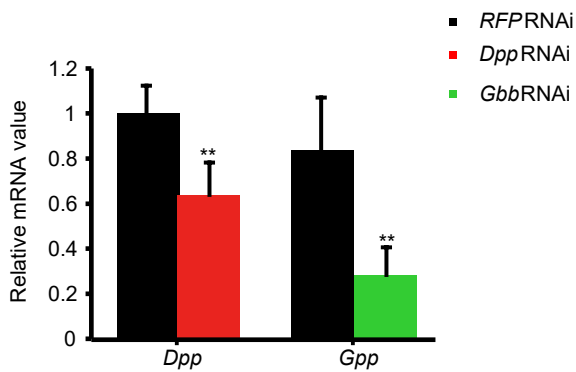

B

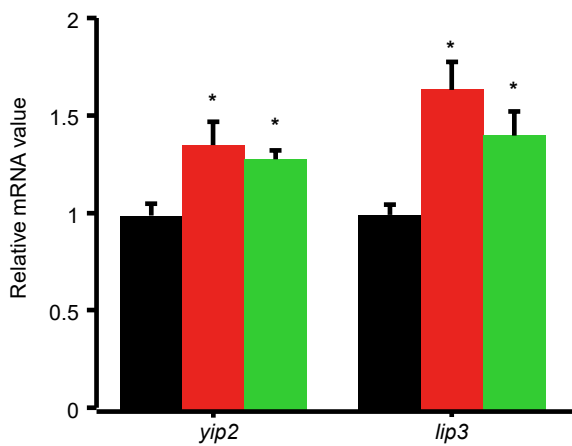

Supplement: Supplementary file 1 [file ijms-19-03525-s001.zip › Figure S3. qRT-PCR analysis of RNAi efficiency and lipase genes.pdf]
